# Productive Infection of Human Skeletal Muscle Cells by Pandemic and Seasonal Influenza A(H1N1) Viruses

**DOI:** 10.1371/journal.pone.0079628

**Published:** 2013-11-05

**Authors:** Marion Desdouits, Sandie Munier, Marie-Christine Prevost, Patricia Jeannin, Gillian Butler-Browne, Simona Ozden, Antoine Gessain, Sylvie Van Der Werf, Nadia Naffakh, Pierre-Emmanuel Ceccaldi

**Affiliations:** 1 Unité Epidémiologie et Physiopathologie des Virus Oncogènes, Institut Pasteur, Paris, France; 2 Unité de Génétique Moléculaire des Virus ARN, Institut Pasteur, Paris, France; 3 UMR 3569, CNRS, Paris, France; 4 Plate-forme de Microscopie Ultrastructurale, Institut Pasteur, Paris, France; 5 UM76, Institut de Myologie, Université Pierre et Marie Curie, Paris, France; 6 U974, INSERM, Paris France; 7 UMR7215, CNRS, GH Pitié Salpêtrière, Paris, France; 8 EA302, Université Paris Diderot, Sorbonne Paris Cité, Paris, France; 9 Cellule Pasteur, Université Paris Diderot, Sorbonne Paris Cité, Paris, France; University of Edinburgh, United Kingdom

## Abstract

Besides the classical respiratory and systemic symptoms, unusual complications of influenza A infection in humans involve the skeletal muscles. Numerous cases of acute myopathy and/or rhabdomyolysis have been reported, particularly following the outbreak of pandemic influenza A(H1N1) in 2009. The pathogenesis of these influenza-associated myopathies (IAM) remains unkown, although the direct infection of muscle cells is suspected. Here, we studied the susceptibility of cultured human primary muscle cells to a 2009 pandemic and a 2008 seasonal influenza A(H1N1) isolate. Using cells from different donors, we found that differentiated muscle cells (i. e. myotubes) were highly susceptible to infection by both influenza A(H1N1) isolates, whereas undifferentiated cells (i. e. myoblasts) were partially resistant. The receptors for influenza viruses, α2-6 and α2-3 linked sialic acids, were detected on the surface of myotubes and myoblasts. Time line of viral nucleoprotein (NP) expression and nuclear export showed that the first steps of the viral replication cycle could take place in muscle cells. Infected myotubes and myoblasts exhibited budding virions and nuclear inclusions as observed by transmission electron microscopy and correlative light and electron microscopy. Myotubes, but not myoblasts, yielded infectious virus progeny that could further infect naive muscle cells after proteolytic treatment. Infection led to a cytopathic effect with the lysis of muscle cells, as characterized by the release of lactate dehydrogenase. The secretion of proinflammatory cytokines by muscle cells was not affected following infection. Our results are compatible with the hypothesis of a direct muscle infection causing rhabdomyolysis in IAM patients.

## Introduction

Influenza viruses are enveloped negative-stranded RNA viruses, members of the *Orthomyxoviridae* family. In humans, they usually cause an acute, self-limited illness, characterized by respiratory and systemic symptoms. The former include nasal congestion, cough and sore throat, and are caused by viral replication and local inflammation in the respiratory tract, while the latter can include fever, headache, fatigue, chills and myalgia [1], and could be due to systemic cytokine responses [2]. 

Besides the classical myalgia, unusual complications of influenza A and B infection can affect skeletal muscles [1]. In children, an acute myopathy following respiratory infection, called "myalgia cruris epidemica" or "benign acute childhood myositis", was first described in 1957 [3]. It is associated with several infectious agents, but occurs predominantly after infection by influenza viruses, mainly of type B [4-7]. Muscle symptoms usually begin a few days after onset of classical influenza symptoms and are typically characterized by bilateral calf pain and tenderness, leading to difficulties with ambulation. The disease is frequently associated with a rhabdomyolysis, as assessed by elevated plasma creatine kinase levels, with rare cases of myoglobinuria. It is more often seen in school-aged boys and is generally self-limited without sequelae [4,7]. In adults, a less characteristic muscle complication may also occur, with generalized weakness and rhabdomyolysis, often leading to myoglobinuria, and predominantly associated with influenza A virus infection [8,9]. Patients with severe rhabdomyolysis can develop a life-threatening acute renal failure [10] or compartment syndrome [11]. 

In April 2009, a new influenza A(H1N1) virus of swine origin appeared in Mexico and rapidly caused a new pandemic as it spread worldwide. Several publications have reported skeletal muscle damage after infection with this pandemic virus. Increased serum creatine kinase (CK) levels were observed in 10 out of 16 patients with pneumonia [12]. Higher CK levels were also measured in non-survivor compared to survivor patients [13], and in critically ill compared to mildly ill patients [14]. Multiple cases of myopathies associated with pandemic influenza in children and adults were also reported [15-24], although the absence of epidemiological studies precludes any conclusion about a possible higher association of this new virus with muscular disease. 

The pathogenesis of influenza associated myopathies (IAM) is poorly known and could be immune-mediated, and/or triggered by direct muscle infection [1] as in alphavirus-induced myositis [25,26]. Although the hypothesis of a “cytokine storm” contributing to the development of IAM is sometimes put forward, levels of proinflammatory cytokines were rarely measured in IAM patients. The data available for one patient with IAM, acute renal failure and myocarditis, show moderate levels of serum proinflammatory cytokines, as observed in most patients with influenza A infection [27]. Rare histological observations from muscle biopsies reveal signs of muscle regeneration together with fiber necrosis, the latter being either moderate and patchy, or more extensive [11,28-36]. Although macrophage infiltration may, rarely, be seen, inflammatory infiltrates are generally absent, thus arguing against the hypothesis of an immune-mediated disease and suggesting that IAM is not a true inflammatory myopathy [1]. Although several attempts at isolating the virus from muscle biopsies have failed [11,28,29,31,33,35-38], A and B influenza viruses have been recovered from muscle biopsy specimens of patients with IAM [30-33,35,39]. In one case, viral particles and viral antigens in muscle cells were also detected *in situ* [30]. More recently, three fatal cases of pneumonia due to the new influenza A H1N1 pandemic virus showed multiple organ invasion with viral particles being detected in the skeletal muscle [40]. 

 In experimentally infected mice, viral RNA was detected in the skeletal muscle of animals inoculated intranasally with an influenza B virus [41], and a non-permissive infection of muscle fibers was reported when the virus was inoculated intramuscularly [42]. These results suggest that IAM could be due to direct infection of muscle cells. Nevertheless, it remains unclear whether influenza viruses can propagate in skeletal muscles. *In vitro* studies on human primary muscle cells report productive infection of differentiated multinucleated myotubes by influenza A(H3N2) viruses [43], by the neurotropic A/WSN/33 strain [44], and by influenza B viruses [45] whereas the susceptibility of undifferentiated myoblasts to infection remains unclear and seems to depend on the viral strain that is being used [43]. One recent publication reports productive infection of a rhabdomyosarcoma cell line by pandemic and seasonal H1N1 influenza viruses [46], but the susceptibility of primary differentiated and undifferentiated human muscle cells to influenza A(H1N1) field isolates has never been studied to date. 

In the present study, we assessed the susceptibility of primary human skeletal muscle cells to two influenza A(H1N1) field isolates representative of the 2009 pandemic (A/California/7/2009) and the 2008 seasonal (A/Paris/1149/2008) viruses. This question was addressed both with undifferentiated, proliferative cells (i. e. myoblasts) and in vitro-differentiated, multinucleated cells (i. e. myotubes). We show that myotubes, and to a lesser extent undifferentiated myoblasts, are susceptible to influenza viruses, and that differentiated myotubes produce an infectious viral progeny. Infection of both undifferentiated and differentiated primary muscle cells leads to cell lysis but has no effect on the secretion of pro-inflammatory cytokines. 

## Results

### Influenza A(H1N1) can infect primary human muscle cells

**Figure 1:**
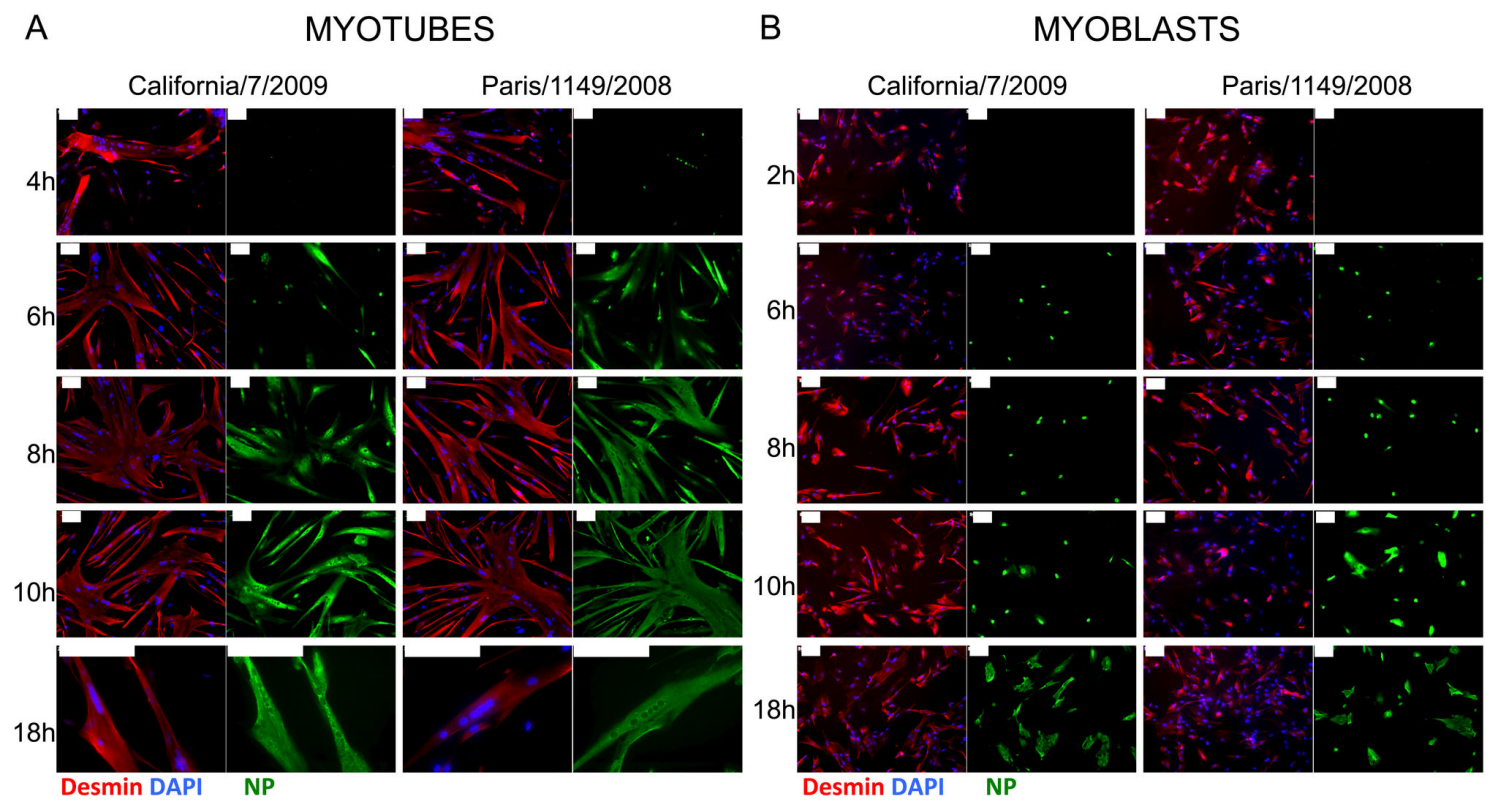
Expression kinetics of the viral nucleoprotein (NP) in infected muscle cells. Indirect immunofluorescence detection of desmin (red, left panels) and NP (green, right panels) in cultures of CHQ donor-derived myotubes (**A**) or myoblasts (**B**) infected by influenza A/California/7/2009 or influenza A/Paris/1149/2008 virus, at various times post-infection. Nuclei were stained with DAPI (blue, left). Bar, 100µm.

Primary human muscle cells, either differentiated or not, were infected at a high multiplicity of infection (MOI) (i.e. 30 pfu/plated cell) with the pandemic A/California/7/2009 and the seasonal A/Paris/1149/2008 influenza A(H1N1) isolates. The expression of the viral nucleoprotein (NP) was then assessed by indirect immunofluorescence staining. Of note, as we did not add TPCK-trypsin in the culture medium, we expected a single cycle of infection to occur. Nuclear NP immunoreactivity was first detected in desmin-positive, multinucleated, differentiated myotubes at 4 hours post-infection (hPI) with the seasonal virus A/Paris/1149/2008, and between 4 and 6 hPI with the pandemic virus A/California/7/2009 (Figure **1A**). NP immunoreactivity was further detected in the nucleus and cytoplasm of myotubes at 6 hPI and afterwards. In undifferentiated, mononucleated myoblasts, nuclear NP immunoreactivity first appeared at 6 hPI (Figure **1B**). Cytoplasmic NP immunoreactivity was detected between 8 and 10 hPI in myoblasts infected with the A/Paris/1149/2008 virus, and after 10 hPI with the A/California/7/2009 virus. At 18 hPI, with both viral isolates, infected myotubes showed a strong and mainly cytoplasmic NP expression, whereas a fraction of infected myoblasts still exhibited a strictly nuclear NP expression. The specificity of the NP staining was checked at 24 hPI in myoblasts and myotubes, using another antibody raised against influenza A(H1N1) (data not shown).

These data demonstrate that influenza A(H1N1) viruses can infect primary human muscle cells *in vitro*, and that the first steps of the viral cycle can take place in both differentiated and undifferentiated muscle cells. Interestingly, the onset of NP expression and nuclear export occurred later in myoblasts than in myotubes. In addition, in myotubes as well as in myoblasts, the time line of NP expression was slightly delayed for the A/California/7/2009 isolate when compared to the A/Paris/1149/2008 isolate, a feature that was also observed in MDCK cells (data not shown).

### Myotubes are highly susceptible to influenza A(H1N1) virus infection

**Figure 2:**
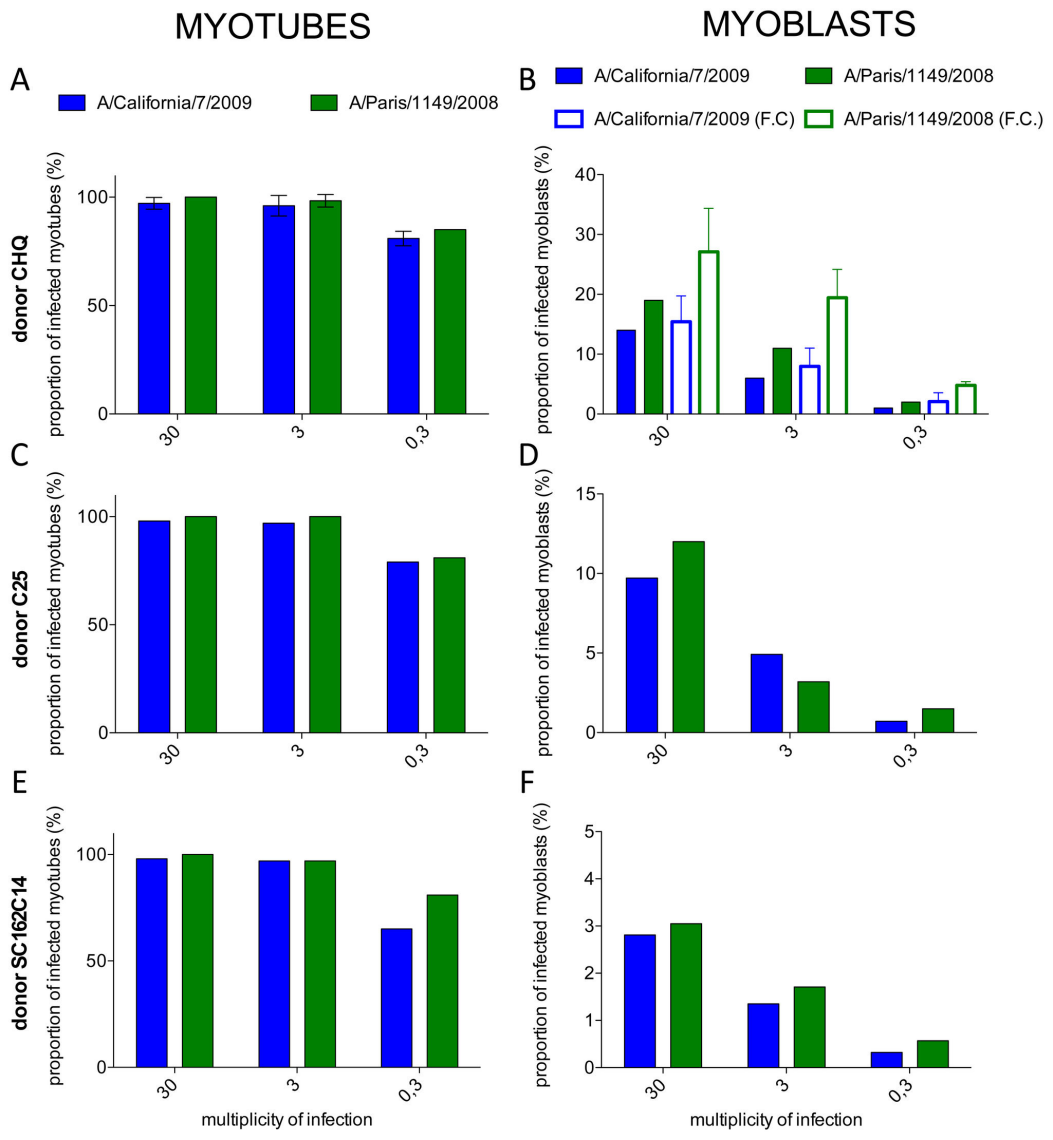
Proportion of infected cells in cultures of myotubes or myoblasts from different donors. **A to F, filled bars**: Indirect immunofluorescence detection of NP and desmin in cultures of muscle cells from three different donors (A, B: CHQ; C, D: C25; E, F: KMSC162C14) and proportion of NP positive myotubes (A, C, E) or myoblasts (B, D, F), 24 hours after infection with the A/Calfornia/7/2009 (blue) or the A/Paris/1149/2008 (green) influenza A(H1N1) isolates. In A, results are the mean of three indenpendent experiments and error bars represent the standard deviation (SD). **B**, **open bars**: Detection of NP and NCAM expression by flow cytometry in cultures of CHQ myoblasts 24 hours after infection by the A/California/7/2009 (blue) or the A/Paris/1149/2008 (green) infuenza A(H1N1) isolates, and proportion of infected, NP-positive cells among NCAM-positive myoblasts. Results are the mean of three independent experiments and error bars represent the SD.

We further assessed the susceptibility of human muscle cells to both influenza A(H1N1) viruses, using various MOIs (0.3, 3 and 30 pfu/plated cells) and muscle cells derived from 3 different healthy donors. We quantified the proportion of cells expressing NP in differentiated myotubes (i. e. cells expressing desmin and containing more than 3 nuclei), and in undifferentiated myoblasts (i. e. mononucleated cells expressing desmin or NCAM), by immunofluorescence and/or flow cytometry, at 24 hPI in the absence of TPCK-trypsin in the culture medium (Figure **2**). Myotubes were shown to be highly sensitive to infection by both isolates, nearly all of them being positively stained for NP at a MOI of 30 or 3, and most of them at a MOI of 0.3 (Figure **2A, 2C** **, 2E**). No differences were seen between the two isolates or between the primary cultures from the three different donors. 

 Myoblasts were less sensitive than myotubes to infection by influenza A(H1N1) viruses since the proportion of NP-positive cells varied from 2.8 to 19% at a MOI of 30 pfu/myoblast, depending on the donor and the viral isolate, and was below 11% at lower MOIs (Figure **2B, 2D** **, 2F**, filled bars). Of note, even at a higher MOI of 300, a high proportion of myoblasts did not express detectable levels of NP (data not shown). 

 Interestingly, myoblasts from the 3 different donors seemed slightly more sensitive to the A/Paris/1149/2008 seasonal isolate than to the A/California/7/2009 pandemic isolate. This trend was confirmed when myoblasts from the CHQ donor were infected at various MOIs and analysed by flow cytometry, using NCAM immunoreactivity to identify myoblasts, and an anti-NP antibody. The proportion of NP-expressing CHQ myoblasts was repeatedly found to be higher with the A/Paris/1149/2008 isolate than with the A/California/7/2009 isolate (Figure **2B** **, open** bars) (p<0.0001, Mann-Whitney U-test). We checked that the differences observed between myotubes and myoblasts were not due to the presence or absence of serum in the culture medium (0% for myotubes vs 20% for myoblasts), by infecting MDCK-SIAT cells with either of the two viruses in the presence of 0, 5 or 20% serum for 6 hours. The proportion of infected cells was then determined by intracellular staining of NP and flow cytometry, and the serum concentration had no effect on the proportion of infected cells (data not shown). Overall, our data indicate that differentiated myotubes are highly susceptible whereas myoblasts are partially resistant to infection by pandemic and seasonal influenza A(H1N1) viruses. 

### Influenza A virus receptors are present at the surface of human muscle cells

**Figure 3:**
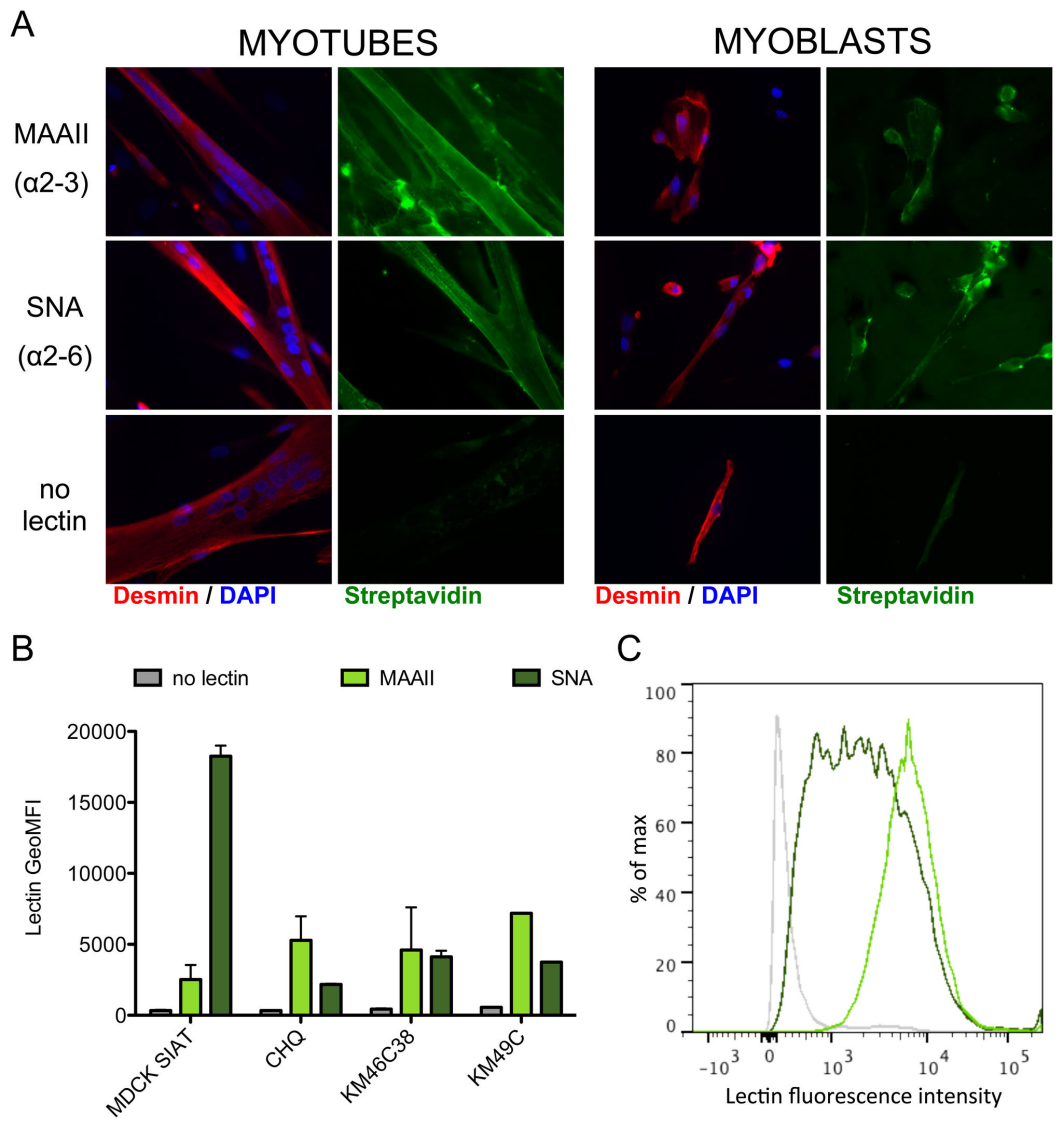
Detection of sialic acids at the surface of human muscle cells. **A**. α2-3 and α2-6 linked sialic acids were detected on non-permeabilized muscle cells (myoblasts and myotubes) from donor CHQ by using the biotinylated lectins MAAII and SNA, respectively. Lectin binding was further revealed by Streptavidin-A488 staining (green), and cells were permeabilized for detection of intracellular desmin (red) by immunofluorescence. **B**. The MAAII and SNA lectins were used for the detection of the two types of sialic acids by cytofluorometry, on non-permeabilized myoblasts from 3 different donors (CHQ, KM46C38, KM49C) and MDCK-SIAT cells as a control. Cells were then permeabilized for the staining of NCAM. The geometric mean of fluorescence intensity (GeoMFI) for each condition (no lectin, grey; MAAII, light green; SNA, dark green) is given for the total population of MDCK cells (which were NCAM-negative) and for the NCAM-positive population in myoblast cultures. Data are the mean of two duplicates with standard deviation as error bar, and represent two independent experiments. **C**. Distribution of the lectin staining intensities in the NCAM-positive population of CHQ myoblasts stained with MAAII (light green) and SNA (dark green).

We asked whether the difference in susceptibility to influenza A virus infection was related to differences in the expression of viral receptors at the surface of myoblasts and myotubes. Indeed, host range and tissue tropism of influenza viruses are in part determined by the specificity of their surface glycoproteins for their receptors, sialic acids attached to galactose by an α2-6 or an α2-3 linkage [47]. Thus, we investigated the presence of α2-6 and α2-3 linked sialic acids on the surface of muscle cells using specific lectins. Both myoblasts and myotubes stained positive with the lectins MAAII (which binds preferentially to α2-3 linked sialic acids) and SNA (which binds preferentially to α2-6 linked sialic acids), as did MDCK-SIAT cells used as a control (not shown) (Figure **3A**). These data suggest that both cells express influenza A virus receptors, and that there are no major differences in the distribution of α2-6 and α2-3 sialic acid receptors at the surface of myoblasts and myotubes. The use of flow cytofluorometry allowed us to confirm that majority of NCAM+ myoblasts from the CHQ donor and two additional donors, KM46C38 and KM49C, were stained with both lectins, as were control MDCK-SIAT cells (Figure **3B and 3C**). Unfortunately, flow cytofluorometry analysis of myotubes is precluded by their large size. However, interestingly, we found that the ratio of α2-6 linked over α2-3 linked sialic acids is lower in myoblasts than in MDCK-SIAT cells, which could contribute to their low susceptibility to human influenza A viruses. 

### Influenza A(H1N1) viruses undergo productive replication in human muscle cells

**Figure 4:**
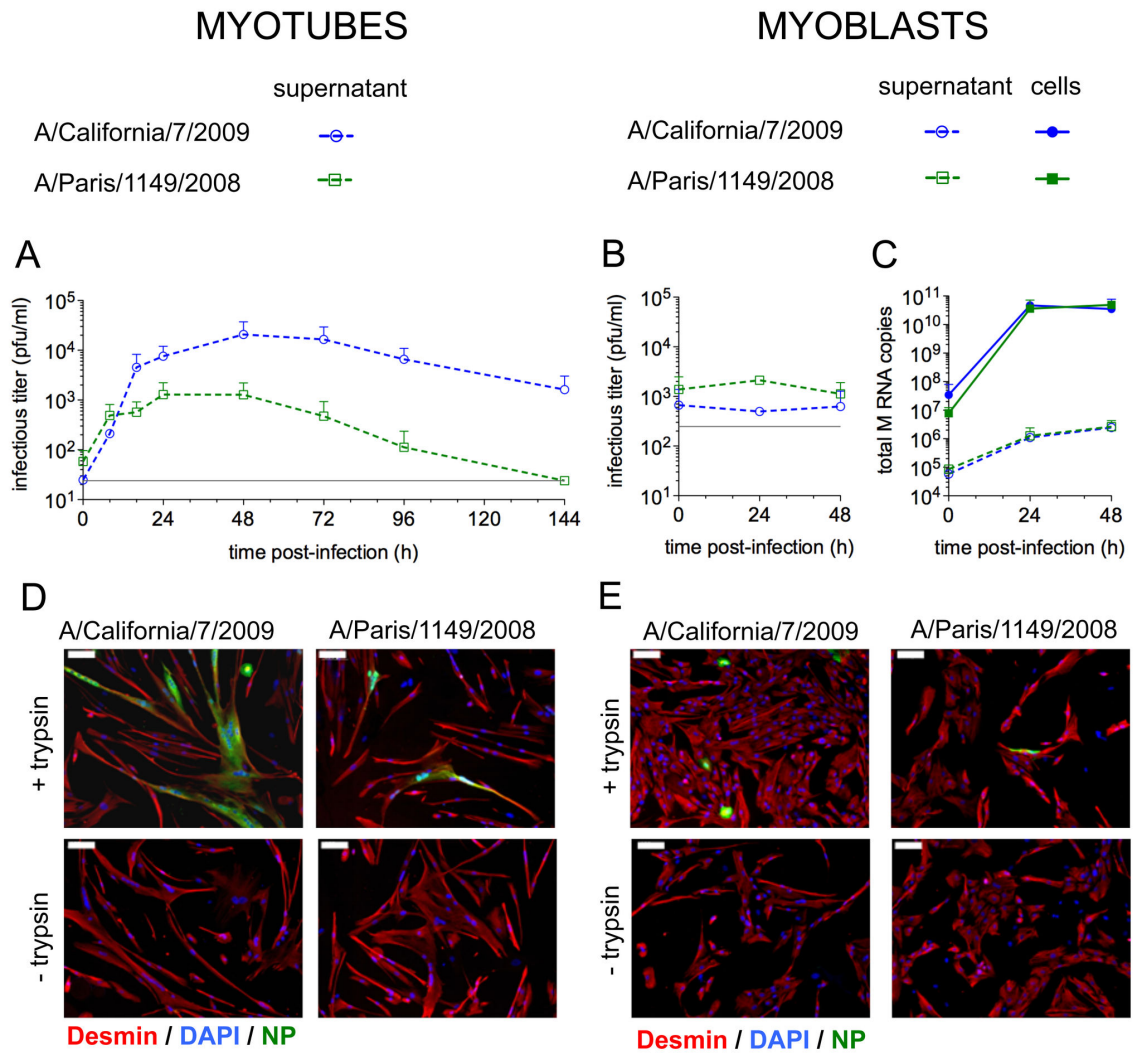
Kinetics of viral RNA and particle production by muscle cells. **A**, **B**. The infectious titers in the supernatants of CHQ myotubes (**A**) or myoblasts (**B**) were measured by plaque assay at various time points after infection with the A/California/7/2009 (blue lines) or the A/Paris/1149/2008 (green lines) viruses. Data are the mean of 2 or 3 independent experiments with the standard deviation. The detection threshold (grey line) was 25 pfu/ml for myotubes and 250 pfu/ml for myoblasts. For this experiment, the concentration of serum in myoblasts’ medium, which could limit the efficiency of viral multiplication and the sensitivity of the plaque assay, was reduced to 5%. **C**. The amount of total M RNAs was quantified by RT-qPCR in supernatants (dashed lines) and in cell extracts (solid lines) of CHQ myoblasts at various time points after infection with the A/California/7/2009 (blue lines) or the A/Paris/1149/2008 (green lines) viruses. Data are the mean of 2 independent experiments with the standard deviation. **D**, **E**. Supernatant from myotubes were harvested 48h after infection with the A/California/7/2009 or the A/Paris/1149/2008 isolates, incubated with or without TPCK-trypsin, and used to infect myotubes (**D**) and myoblasts (**E**) from the same donor (CHQ). Indirect immunofluorescence detection of desmin (red) and NP (green) and staining of the nuclei with DAPI (blue) were performed. Bar: 100µm.

We examined whether influenza A viruses could undergo productive replication following infection of muscle cells at a MOI of 0.3 pfu/plated cell, by measuring the infectious titer in the supernatants of infected cells at various time points (Figure **4A, 4B**). To allow the maturation of the viral hemagglutinin HA, samples were treated with 1µg/ml TPCK-trypsin before titration by plaque assay. This treatment was sufficient to allow complete maturation of both viruses, as assessed by testing increasing concentrations of trypsin (data not shown). In the supernatants of myotubes (Figure **4A**), the infectious titers increased as early as 8 hPI, and reached a maximum 48h PI before decreasing, probably due to cell death and degradation of virions. The pandemic virus was produced at higher titers than the seasonal virus. 

 In the supernatants of myoblasts (Figure **4B**), we did not observe any increase in the viral titer following infection. However, the presence of serum in myoblast cultures decreases the sensitivity and accuracy of the plaque assay, possibly masking low variations in viral production. Thus, we extracted RNA from supernatants and from the corresponding cells to quantify the production of viral genomic and messenger RNAs from the M segment (Figure **4C**). In supernatants, the amounts of M RNAs increased moderately with time for both isolates. However, the sharp accumulation of M RNAs observed in cellular extracts for both isolates indicated that influenza viruses undergo no major transcription/replication defect in myoblasts. 

 To determine whether the viral particles produced by myotubes were able to infect muscle cells, the supernatants of CHQ myotubes collected at 48 hPI were used to infect myotubes and myoblasts derived from the same donor. When the viral inoculum was pre-treated with TPCK-trypsin, NP expression was detected 24 hours later in muscle cells (Figure **4D, 4E** **, + trypsin**). In contrast, in the absence of protease treatment, muscle cells were not infected (Figure **4D, 4E** **, -trypsin**), which indicates that the HA is not efficiently cleaved in cultures of myotubes.

### Viral budding occurs in myotubes and in myoblasts

**Figure 5:**
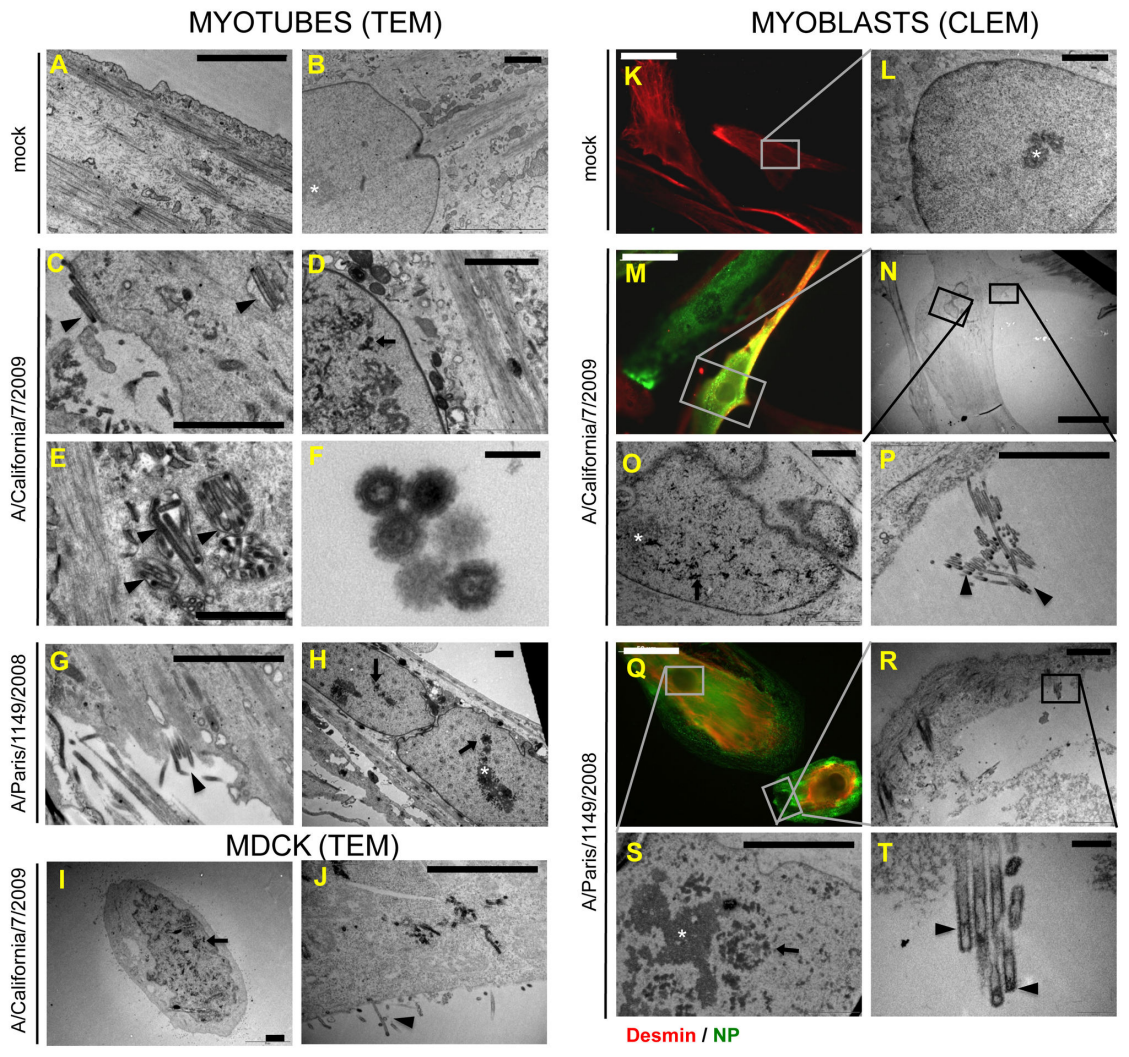
Virus budding from infected myotubes and myoblasts. **A to J**. Transmission electron microscopy (TEM) was performed on non-infected myotubes (**A**, **B**), on myotubes infected for 20h with the A/California/7/2009 (**C**, **D**, **E**, **F**) or the Paris/1149/2008 (**G**, **H**) isolates, and on MDCK-SIAT cells, 16h after infection with the A/California/7/2009 isolate (**I**, **J**). Arrowheads: budding virions or virions in close contact with the cell. Arrows: dense nuclear aggregates. Stars: nucleoli. Bars: A to E and G to J: 2µm; F: 100nm. **K to T**. Correlative light and electron microscopy (CLEM) was carried out on non-infected myoblasts (**K**, **L**) and on myoblasts infected for 20h with the A/California/7/2009 (**M**, **N**, **O**, **P**) or the A/Paris/1149/2008 (**Q**, **R**, **S**, **T**) isolates. **K**, **M**, **Q**. Immunofluorescence imaging of myoblasts expressing desmin (red) and/or NP (green). **L**. Transmission electron microscopy imaging of a non-infected myoblast observed in K showing an intact nucleus. **N**, **O**, **P** and **R**, **S**, **T**. Transmission electron microscopy imaging of the cells observed in M and Q, respectively, showing budding virions or nuclear aggregates. Arrowheads: budding virions or virions in close contact with the cell. Arrows: dense nuclear aggregates. Stars: nucleoli. Bars: K, M, Q: 50µm; N, R: 20 µm; L, O, P, S: 2µm; T: 200nm.

The low infectivity and M RNA quantity in supernatants from infected myoblasts prompted us to check whether viral budding occurred in muscle cells, by means of transmission electron microscopy (TEM), 20 hours after infection at a MOI of 30 pfu/plated cell. In myotubes, we were able to observe both filamentous and spherical extracellular and budding virions, mostly at the cell surface, that were absent in non-infected cell samples (Figure **5A, 5C** **, 5G, arrowheads**.). Virions were also rarely observed in seemingly intracellular vesicles, which are most likely extracellular spaces between the cells and the surface of the culture plate, due to the local invagination of the plasma membrane (Figure **5C, 5E** **, arrowheads**). These particles exhibited the typical morphology of influenza viruses: a diameter of 80 to 100 nm and spikes (Figure **5F**), and for some of them, a dense area at one pole that suggested the presence of ribonucleoparticles (RNPs). Most analyzed infected cells presented nuclear inclusions of dense material that were never observed in uninfected cells (Figure **5B, 5D** **, 5H, arrows**) and were distinct from nucleoli (Figure **5B, 5D** **, 5H, stars**). Virions and nuclear aggregates were also observed in control cultures of MDCK-SIAT infected with the A/California/7/2009 virus (Figure **5I and 5J**). 

Using the same technique, only rare virions could be observed in myoblast cultures (data not shown). Thus, we conducted correlative light and electron microscopy (CLEM) to specifically observe desmin-positive and NP-expressing myoblasts and distinguish myoblasts from the small fraction of desmin-negative cells that are also present in the culture. We found extracellular virions close to several NP-expressing myoblasts as well as around NP-expressing, desmin-negative cells. Most of the studied NP-expressing myoblasts exhibited budding virions, or virions trapped between the cell and the culture stand (Figure **5M, 5N**, **5P**, **5Q**, **5R**, **5T** **, arrowheads**). In addition, nuclear aggregates identical to those seen in myotubes and in infected MDCK-SIAT cells were also detected in nearly all infected myoblasts (Figure **5O, 5S** **, arrows**) but not in uninfected ones (Figure **5L**). 

A total of 31 individual cells were screened using this technique, and the frequency of extracellular virions, budding events and/or nuclear aggregates was assessed for 11 and 15 NP-expressing cells for the pandemic and seasonal viruses, respectively, as well as 5 NP-negative control cells (Table **1**). Overall, 55 to 60 % of analysed infected cells exhibited viral budding at their membrane and 100% presented at least one feature of infection. From our data we conclude that not only myotubes, but also myoblasts, are able to yield influenza A(H1N1) virions.

**Table 1:** Summary of infection features observed in myoblasts by CLEM.

Virus	IF status	N	Number (proportion) of cells with			
			budding virions	extracellular virions	aggregates in nucleus*	one of these features
A/California/7/2009	Desmin+ NP +	11	6 (55%)	6 (55%)	8/8 (100%)	11 (100%)
	Desmin+ NP-	1	0	0	0	0
A/Paris/1149/2008	Desmin+ NP +	15	9 (60%)	4 (27%)	12/13 (92%)	15 (100%)
	Desmin+ NP-	1	0	0	0	0
mock	Desmin+ NP-	3	0	0	0	0

* the nucleus could not be observed for all studied cells, the number of cell whose nucleus was seen is thus indicated and the proportion of positive cells is based on this number.

### Influenza A(H1N1) infection of myotubes and myobasts leads to cell lysis

**Figure 6:**
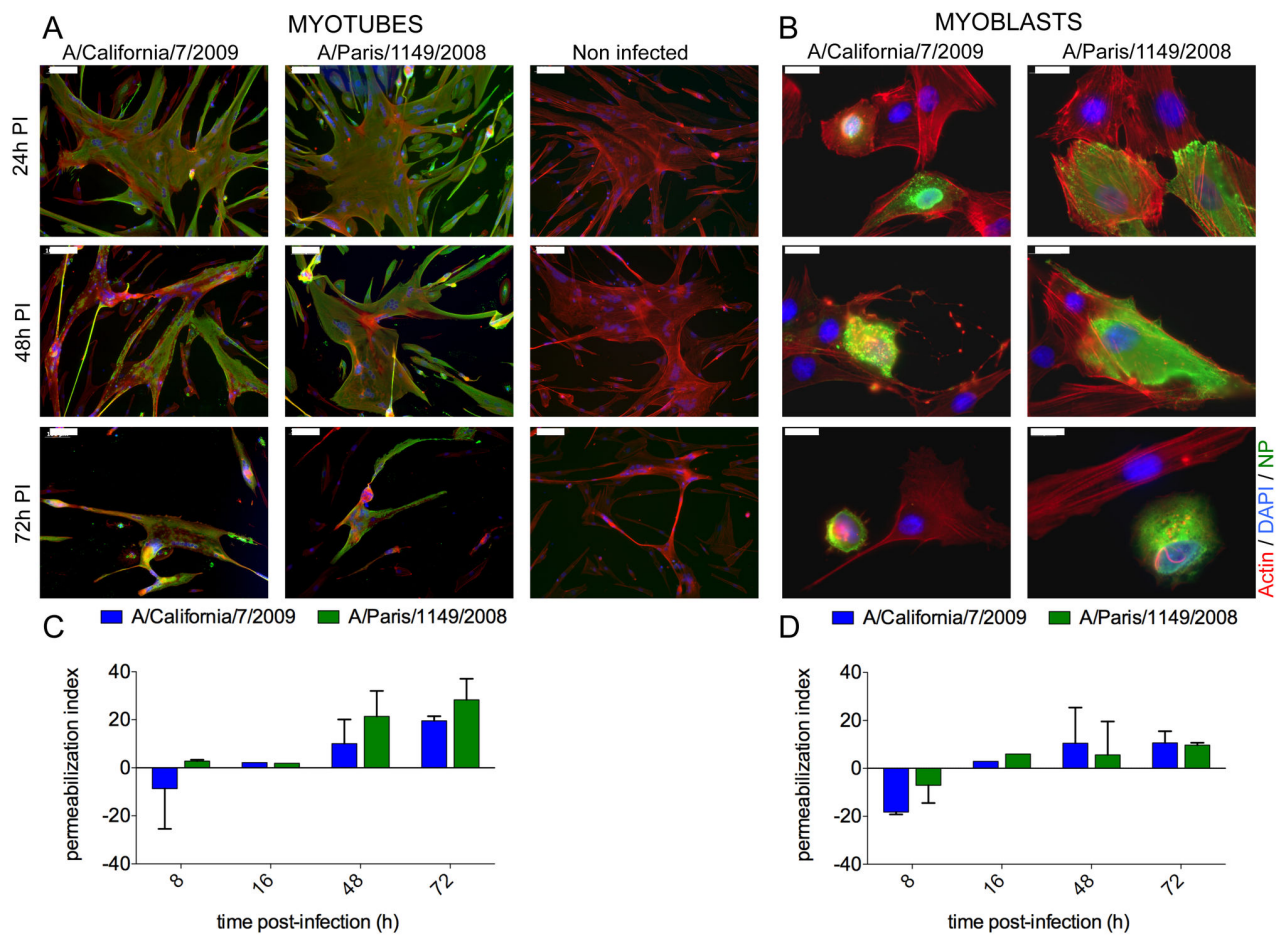
Muscle cell death and lysis after infection by influenza A(H1N1). **A**, **B**. The morphology of the actin cytoskeleton (red) of myotubes and myoblasts (donor CHQ) was observed by immunofluorescence at various time points after infection by the A/California/7/2009 or A/Paris/1149/2008 isolate and in non-infected cultures grown in parallel. The nucleoprotein NP (green) was used to discriminate between infected and non-infected cells, and DAPI was used to stain the nucleus. Bars: A, 100µm, B, 20µm. **C**, **D**. The concentration of lactate dehydrogenase (LDH) released in the culture supernatants was measured by an enzymatic assay at various time points after infection by the A/California/7/2009 or the A/Paris/1149/2008 isolate. This concentration was normalized using untreated and chemically-permeabilized non-infected cultures to calculate a rate of LDH release reflecting the index of cell permeabilization. Results are expressed as the mean and SD of two independent experiments on CHQ cells.

Myoblasts and myotubes cultures were infected at a high MOI of 30 pfu/plated cell and cell morphology was further assessed by indirect immunofluorescence at various time points after infection (Figure **6A, 6B**). In cultures of infected myotubes, NP-expressing cells started shrinking and detaching at 48 hPI, while non-infected myotubes degenerated after 72 hPI. The NP immunoreactivity pattern became irregular and patchy in most infected myotubes at 48 hPI, but the actin cytoskeleton was not altered (Figure **6A**). In infected myoblasts, NP-expressing cells also started rounding and detaching at 48 hPI, and this was accompanied by a disruption of the actin cytoskeleton and a loss of stress fibers, whereas no nuclear alterations were seen (Figure **6B**). Uninfected, NP-negative cells did not exhibit any morphological changes during the same period. 

In parallel, we measured the amount of lactate dehydrogenase (LDH) in the culture supernatant to quantify cell lysis and calculate a cell permeabilization index. This index began to increase at 48 hPI in cultures of myotubes infected by the seasonal virus (Figure **6C**, green bars), at 72 hPI in cultures of myotubes infected by the pandemic virus (Figure **6C**, blue bars) and at 72 hPI in cultures of myoblasts for both viral isolates (Figure **6D**). Thus, the cytopathic effect observed by immunofluorescence was followed by an increase in cell permeability, indicating that infection of myotubes and myoblasts by influenza A(H1N1) viruses leads to cell lysis. 

### Infection does not affect the secretion of cytokines by muscle cells

**Figure 7:**
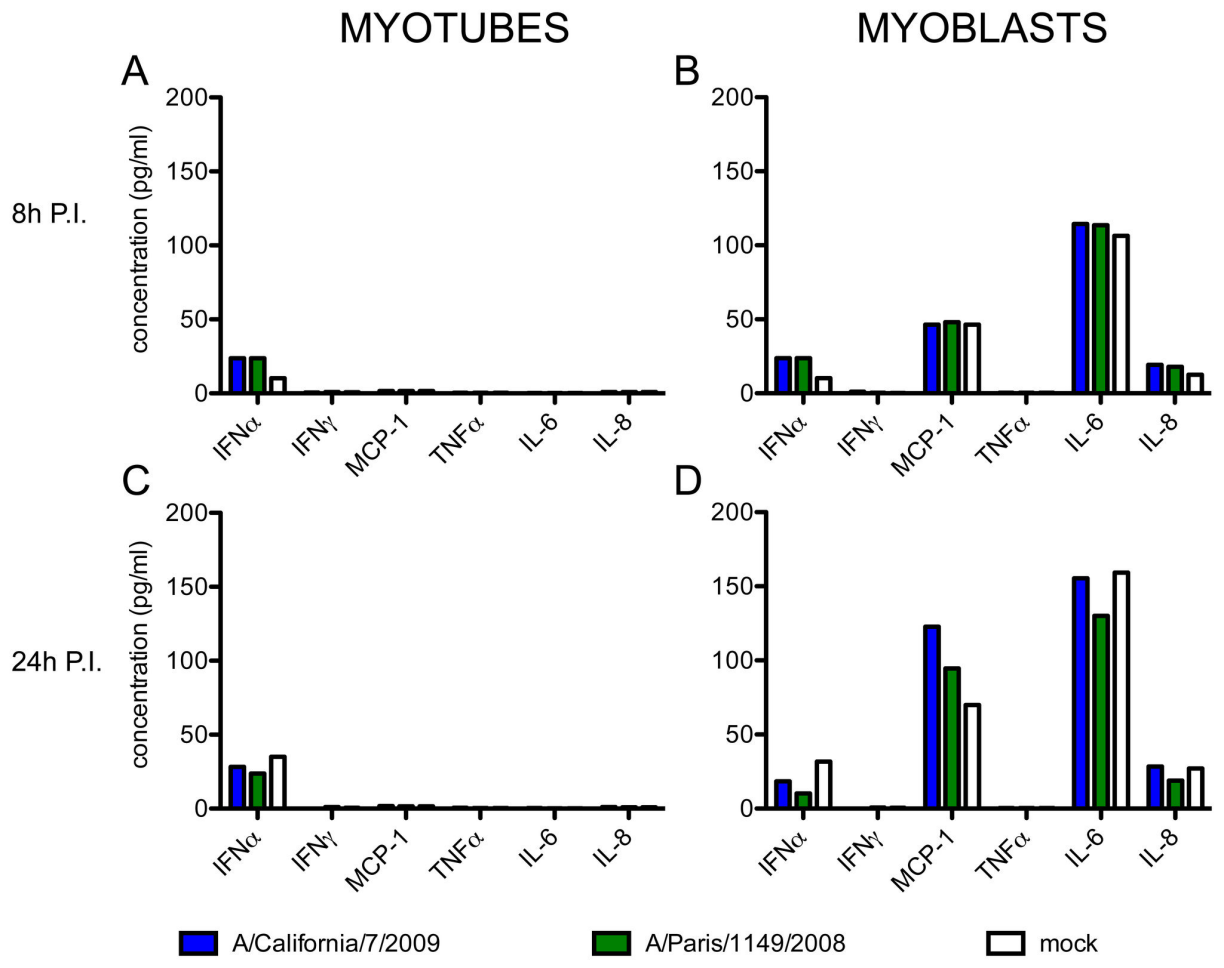
Secretion of 5 cytokines and 1 chemokine by infected muscle cells. The concentrations of IFNα, IFNγ, TNFα, IL-6 and IL‑8 and MCP-1 were simultaneously measured in the culture supernatants of myotubes (left panels) and myoblasts (right panels), at 8h (**A**, **B**) and 24h (**C**, **D**) after infection with the influenza viruses A/California/7/2009 (blue bars) or A/Paris/1149/2008 (green bars), or in non-infected cells cultured in parallel (white bars). These results are representative of two independent experiments on cells from donor CHQ.

We assessed the concentration of 5 cytokines (IFNα, IFNγ, TNFα, IL-6, IL-8) and 1 chemokine (MCP-1) in the supernatant of cultured myoblasts or myotubes at 8 hPI (Figure **7A, 7B**) and 24 hPI (Figure **7C, 7D**). In the supernatants of myotubes, whether infected or not, all proteins were undetectable except IFNα, whose levels were just above the detection threshold in all conditions (Figure **7A, 7C**). High levels of MCP-1, IL-6 and moderate levels of IL-8 were detected in the supernatant of mock-infected myoblasts at 8 hPI, and even more so at 24 hPI (Figure **7B, 7D** **, white bars**), which probably corresponded to the accumulation of these proteins over time, in agreement with the literature [48]. IFNα, TNFα and IFNγ remained below or just above the detection threshold. Infection by the pandemic or the seasonal influenza A(H1N1) viruses had no effect on the secretion of the tested proteins (Figure **7B, 7D**, blue and green bars) .

## Discussion

Several hypotheses may explain the development of an acute myopathy associated with influenza virus infection (IAM). Among them, the direct infection and lysis of muscle cells [1,49] is supported by the isolation of influenza viruses from IAM patients’ skeletal muscle samples, or the observation of viral particles in muscle tissue [30-33,35,39,40]. Extra-pulmonary replication of influenza viruses is rarely reported in infected individuals, but viremia is sometimes detected even in patients with mild disease and was found in pandemic influenza A (H1N1) patients with acute illness and with elevated CK levels [49]. Besides, influenza viruses seem to replicate in the myocardium during influenza-induced myocarditis [11], and viral particles were detected in the skeletal muscle of three fatal cases of pneumonia with multiple organ invasion due to the influenza A (H1N1) pandemic virus [44]. Previous work on animal models and muscle cell cultures suggested that influenza viruses could indeed infect the skeletal muscle, but with conflicting results about the productivity of infection and the induction of a local inflammation [42,43]. 

To further assess the susceptibility of human skeletal muscle to influenza A(H1N1) viruses and to examine whether it is modulated upon differentiation of myoblasts into myotubes, we took advantage of primary cultures of human muscle cells that are able to differentiate *in vitro*. This model has been recently used in our laboratory to study skeletal muscle infection by the Chikungunya virus, where it recapitulated observations made on muscle biopsies from virus-infected patients [26]. Previous studies using primary cultures of muscle cells suggested a productive infection of differentiated myotubes by influenza B and influenza A(H3N2) viruses, and by the neurotropic A/WSN/33 strain [43-45]. The susceptibility of myoblasts to infection and their ability to produce infectious virions remains unclear [43], mostly because it was not explored with recent, more sensitive techniques. Here, we combined recent molecular and imaging techniques to address these questions. We also took advantage of early passages of influenza A(H1N1) field isolates to avoid a possible effect of cell culture adaptation on viral tropism. 

Using muscle cells from three different donors, we found that myotubes are highly susceptible to infection with influenza A(H1N1) viruses (Figure **2**), in agreement with the detection of α2-6 and α2-3 linked sialic acids at their surface (Figure **3**). The increase of infectious titers in the supernatants from myotubes (Figure **4**) taken together with the detection by means of transmission electron microscopy (TEM) of numerous budding and extracellular virions (Figure **5**), strongly suggest that mature infectious virions are produced from infected myotubes. We also showed that infectious viruses produced by myotubes require TPCK-trypsin treatment to further infect muscle cells (Figure **4**). Thus, propagation of influenza viruses in the muscle tissue would require a local expression of adequate proteases for the maturation of HA. Plasminogen-derived plasmin, which is able to cleave HA [50], could facilitate influenza virus propagation in the muscle, as plasminogen is ubiquitous and the urokinase-type plasminogen activator is expressed by muscle cells during regeneration [51]. The local induction of expression of other proteases upon infection, as observed in the myocardium during influenza-associated myocarditis [52], could also favour viral propagation. To address these questions, identification of active proteases in muscle biopsies from IAM patients would be required.

Unlike myotubes, myoblasts appeared partially resistant to infection with influenza A(H1N1) viruses. As myoblasts are smaller in size, they are less likely than myotubes to encounter a viral particle, which could reduce their susceptibility to infection. However, the mean number of nuclei per myotubes being around 7 in our cultures (data not shown), the difference in size between myoblasts and myotubes cannot fully account for the large differences in the proportions of infected cells, which are more likely due to the differentiation state. The low proportion of NP-expressing myoblasts, even at high MOIs, suggests that an early blockade, at the step of viral entry or post-entry, is restricting the replication of influenza A(H1N1) viruses in a subpopulation of these cells, in agreement with an earlier study [43]. The tissue tropism and host range of influenza viruses is determined in part by the receptor specificity of their surface glycoproteins [47].. It is commonly accepted that human influenza A viruses bind preferentially to sialic acids attached to galactose by an α2-6 linkage, although recent studies point to the wide diversity of α2-6 and α2-3 linked sialic acids at the surface of the human respiratory tract epithelium, and to the complexity of the determinants of influenza virus efficient binding [53]. To our knowledge, the presence of α2-3 or α2-6 linked sialic acids on the surface of muscle cells has not been assessed up until now. Here we detected both α2-6 (bound by SNA lectin) and α2-3 (bound by MAAII lectin) linked sialic acids at the surface of myoblasts (Figure **3**). In an attempt to compare more precisely the distribution of influenza virus receptors on myotubes versus myoblasts, we used serial dilutions of the lectins. We could not find an experimental condition in which one cell type was stained while the other was not, following serial dilutions (data not shown), which suggest that there were no major differences in the levels of α2-6 and α2-3 linked sialic acids present at the surface of myoblasts and myotubes. In cytofluorometry, the distribution of lectin staining on NCAM+ myoblasts was monomodal without a separate negative population (Figure **3C**). This does not rule out that the sub-population of cells with lower α2-6 and/or α2-3 sialic acid expression might be resistant to infection at the entry level [53]. Overall, the heterogeneity of susceptibility to influenza A H1N1 viruses in myoblast population remains to be understood, and could be related to different states of proliferation, activation or differentiation. 

Although some myoblasts did express NP after infection, they showed several differences when compared to infected myotubes. First, the onset of NP expression and nuclear export occurred later in myoblasts than in myotubes. Second, myoblasts with a strictly nuclear NP immunoreactivity were detected at 18 hPI and at later time points, whereas NP staining was mostly cytoplasmic in myotubes at this late time point. Third, no increase in infectious titers could be measured and only a low increase in extracellular M RNA levels was detected in myoblasts’ supernatants, although cell-associated M RNA levels did increase. Correlative light and electron microscopy (CLEM) demonstrated that most NP-expressing myoblasts showed signs of virion budding (Table **1**, Figure **5**). However, as > 40% of such cells did not exhibit viral budding, and as budding virions may not be infectious, a partial defect in the late steps of the viral cycle cannot be ruled out. Overall, our observations suggest that several distinct blockades of the viral cycle, not only at an early step of the viral cycle but also possibly at the step of RNP nuclear export and/or at the step of virion assembly or budding, could impair the production of a mature viral progeny by myoblasts. 

In previous studies, influenza A or B virus infection of muscle cells led to a cytopathic effect with release of intracellular content 2 days PI [43,45], which was also observed in the present study (Figure **6**). Although influenza A viruses are usually pro-apoptotic, infected muscle cells exhibited necrotic rather than apoptotic features (Figure **6**), consistently with their known resistance to apoptosis [54] and with the rhabdomyolysis and fiber necrosis observed in IAM patients [30,32]. We also show that infection of muscle cells with influenza A viruses does not induce the secretion of pro-inflammatory cytokines (Figure **7**). In particular, the constitutive secretion of IL-6, MCP-1 and IL-8 by myoblasts, which was previously described [48] and was confirmed in our experiments, was not affected by influenza A virus infection. Our *in vitro* model seems to recapitulate the *in vivo* findings of muscle necrosis and low inflammation in IAM biopsies, and suggests that a direct cytopathic effect of influenza virus infection could trigger the rhabdomyolysis observed in affected patients. However, the existence of other pathogenic mechanisms cannot be ruled out. Indeed, quiescent satellite cells and fully matured fibres may respond differently to influenza A virus infection than cells in our *in vitro* model, which consists of proliferating myoblasts and multinucleated myotubes mimicking those found in a regenerating muscle. Interestingly, previous studies in the murine model suggested that induction of muscle regeneration might increase the susceptibility of the muscle tissue to influenza viruses [44,55]. Further understanding of the pathogenesis of IAM will require combined analysis of muscle biopsies from affected patients and their local and systemic immune status. 

## Materials and Methods

### Cells and viruses

Human muscle cells (myoblasts) from three different healthy donors were obtained from the AFM cell bank (Association Française contre les Myopathies – Généthon). They were isolated as previously described [56], in accordance with the French legislation on bioethics, and with informed consent of the patient or his legal representative. Primary myoblasts were originally isolated from the quadriceps of a 5-day-old infant (CHQ), a 14-year old boy (KMSC162C14), a 25-year old man (C25), a 38-year old man (KM46C38) and a 49-year old man (KM49C). Myoblasts in primary culture were grown in F10-Ham with GlutaMax (Gibco) supplemented with 20% heat-inactivated foetal bovine serum (FBS, Gibco) and gentamycin (50µg/ml, Euromedex). To allow their differentiation into myotubes, myoblasts were washed twice with Dulbecco modified Eagle's medium (D-MEM, Gibco) supplemented with GlutaMax and gentamycin, and then grown in differentiation medium, i.e. D-MEM with GlutaMax, gentamycin, insulin (10µg/ml, Sigma) and transferrin (100µg/ml, Sigma). MDCK-SIAT cells were grown in modified Eagle's medium (MEM, Gibco) supplemented with gentamycin, glutamine (2mM), tricine (8mM) and 5% FBS.

The influenza viruses A/California/07/2009, kindly provided by Alan Hay (NIMR, London, UK), and A/Paris/1149/2008, isolated by the National Reference Center for influenza (Northern France), were amplified by two serial passages at a multiplicity of infection of 10^-4^ pfu/cell on MDCK cells. They were titrated using a plaque assay adapted from Matrosovitch et al. [57] on MDCK-SIAT cells.

### Infections

For immunofluorescence, electron microscopy and viral production experiments, myoblasts were plated at a density of 15.10^3^ cells / cm^2^, on coverslips in 24-wells plates or on 6-wells plates. For measurement of lactate dehydrogenase (LDH) or cytokine release, myoblasts were plated at a density of 20.10^3^ cells / cm^2^ on flat-bottom 96-wells plates. For cytofluorometry, myoblasts were plated at a density of 20.10^3^ cells / cm^2^ on 6-wells plates. To obtain myotubes, myoblasts were plated at the same density, left in their medium for 2 days and then incubated in differentiation medium for 5 to 6 days. When used for culture of myotubes, coverslips were previously coated with collagen type 1 (Rat tail, BD). Multiplicity of infection (MOI) was calculated as the number of plaque forming units (pfu, as assessed on MDCK cells) per initially plated myoblast. Myoblasts were infected one day after plating, and myotubes after 5 or 6 days of differentiation. Before infection, cells were washed twice with phosphate-buffered saline (PBS) to remove serum. Virus was added on cells in a minimal volume of D-MEM supplemented with gentamycin without serum. After one hour of incubation, cells were washed with PBS and culture medium not supplemented with TPCK-trypsin was added. 

### Immunofluorescence staining and light microscopy

Cells were fixed in PBS containing 4% paraformaldehyde (Sigma) and washed in PBS. Saturation and permeabilization were performed in PBS with 10% normal goat serum (Vector) and 0.1% Triton X-100 (Sigma). Coverslips were incubated with the following dilutions of primary and secondary antibodies: anti-NP mouse IgG2b, 1/200 (Argene) and FITC-coupled anti-mouse Ig, 1/100 (Vector). For actin staining, a 1/300 dilution of A567-coupled phalloidin (Invitrogen) was added to the solution of secondary antibody. For desmin staining, coverslips were incubated in a 1/100 dilution of anti-desmin rabbit polyclonal antibody (Abcam) overnight at 4°C, and then in a 1/1000 dilution of Alexa-547 coupled anti-rabbit IgG secondary antibody (Pierce). For co-staining of NP and influenza A H1N1 antigens, cells were permeabilized in PBS with 0.1% Triton X-100 and saturated with 2.5% normal horse serum. Coverslips were incubated in a 1/100 dilution of anti-influenza A(H1N1) virions goat polyclonal antibody (Abcam) and then in a 1/100 dilution of donkey anti-goat Ig (Vector). The preparation was further fixed in 4% paraformaldehyde (Sigma), and further saturated with 10% normal goat serum. Then, immunostaining of NP and desmin were conducted as already described. Coverslips were finally mounted in DAPI-Fluoromount G (Southern Biotech) on Superfrost-plus microscope slides (Thermo Scientific). Images were acquired and analysed with a Zeiss AxioPlan fluorescent microscope with a Zeiss AxioCam camera and analysed with the Zeiss AxioVision software. 

### Cytofluorometry

At 24 hours post-infection (hPI), myoblasts were washed with PBS-EDTA 1%, incubated in PBS-EDTA 1% at 37°C, harvested and fixed in PBS with 1% paraformaldehyde (Sigma). After PBS washing, cells were further incubated in 50µl PBS containing 1% bovine serum albumin (BSA) and 0.05% saponin, anti-NCAM polyclonal goat antibody 1/10 (R&D Systems) and anti-NP monoclonal mouse antibody 1/200 (Argene) and then in 50µl PBS, 1% BSA, 0.05% saponin with APC-coupled anti-goat IgG antibody, 1/25 (R&D Systems) and FITC-coupled anti-mouse IgG, 1/100 (Vector). Samples were assayed using a FACSCalibur cytofluorometer (Beckton-Dickinson) and results were analysed using CellQuest and FlowJo softwares.

### Detection of sialic acids

MDCK and myoblasts were plated the day before, and myotubes were put to differentiate for 5 days before staining. Cells were washed once in PBS containing 1% FBS, and incubated live for 1h at 4°C in PBS, FBS 1% with biotinylated *Maackia Amurensis* lectin II 1/100 (MAAII, Vector Laboratories) or biotinylated *Sambucus Nigra* lectin 1/200 (SNA, Vector Laboratories). Cells were washed once in PBS, fixed in PFA 4%, washed and incubated in PBS FBS 1% with 5μg/ml of Streptavidin-A488 (Life Technologies), for 1h at 4°C. Preparation were then processed for desmin staining as described above and were image using a Leica DM6000B microscope with a Photometrics CoolSNAP25 HQ2 camera controlled by the MetaMorph software. Images were processed using the ImageJ software. 

 For relative quantification of sialic acid expression by FACS, myoblasts and MDCK were plated the day before staining at 200.10^3^ cells/well in 6-well plates. Cells were detached by incubation in PBS EDTA 1mM for 15 min. at 4°C, washed in PBS and PBS FBS1% and incubated for 1h at 4°C in PBS FBS 1% with MAAII 1/100 or SNA 1/200. Cells were then washed 2 times in PBS FBS 1%, and incubated for 1h in PBS FBS 1% with 5μg/ml of Streptavidin-A488. After 2 washes in PBS FBS 1%, cells were fixed in PFA 4% and further processed for NCAM staining as described above. Samples were assayed using a Verse cytofluorometer (Beckton-Dickinson) and results were analysed using the FlowJo software.

### Transmission electron microscopy

Myotubes and myoblast cultures were processed for electron microscopy as described previously [58]. Briefly, cells were fixed at 20 h PI in 2.5% glutaraldehyde, 0.1M cacodylate buffer overnight at 4°C and post-fixed in 1% osmium tetroxide, 0.1M cacodylate buffer. Samples were dehydrated through a graded ethanol series and incubated overnight in a mixture of 50% ethanol and then incubated in pure resin Epon and polymerized for 48h hours at 60°C. Ultrathin sections (70–80 nm) were performed with an ultramicrotome Leica UCT, stained with uranyl acetate and Reynold’s lead citrate, and then observed at 80 kV accelerating voltage in a JEOL 1200EXII electron microscope equipped with an Eloïse Mega View III camera. 

### Correlative light and electron microscopy

Myoblasts and MDCK-SIAT cells were plated onto cell-culture plates containing a glass insert with a grid (MatTek Corporation) at a density of 10.10^3^ cells/cm^2^. The following day, they were infected at MOI of 30 pfu/myoblast using the protocol described before. After 20h for myoblasts, and 16h for MDCK-SIAT, cells were fixed in 3% paraformaldehyde (Sigma), 0.05% glutaraldehyde PBS for 30 minutes at room temperature. Samples were permeabilized and saturated for 15 minutes in PBS containing 0.2% bovine serum albumin (Sigma) and 0.05% saponin (Sigma). Samples were incubated in a mix of primary antibodies against NP (mouse, Argene) and desmin (rabbit, Abcam) for 2 hours in PBS containing 0.2% bovine serum albumin and 0.01% saponin, then in a mix of secondary antibodies against mouse (Vector) and rabbit (Pierce) in the same buffer for 45 minutes. After washing, samples were kept in PBS and imaged with an inverted Zeiss AxioPlan microscope with a Zeiss AxioCam and the Zeiss AxioVision software. Following fluorescent light microscopy, samples were fixed and processed for electron microscopy as described earlier.

### RNA extraction and quantitative RT-PCR

At each time point of the kinetics, supernatants from myoblasts were removed and clarified by centrifugation (500g, 5 min) while cells were washed and harvested after incubation in Trypsin-EDTA (Invitrogen) and subsequent centrifugation (500g, 5 min). RNA was extracted from cellular and supernatant samples using the RNeasy Mini kit (Qiagen). Cells were lysed with 350µl of lysis buffer, whereas 150µl of lysis buffer were added to 200µl of supernatant samples. Subsequent steps were performed following manufacturer's instructions, using a final elution volume of 60µl. 

 Taq-Man quantitative RT-PCR was performed using the SuperScript III Platinum OneStep qRT-PCR system (Invitrogen), using 2.5µl of RNA (standard or RNA extracted form samples either undiluted or at a 1/100 dilution), with a final reaction volume of 10µl, in a LigthCycler 480 thermocycler (Roche). The following primers were used for specific amplification of a 155 bp portion of M viral RNAs: CTTCTAACCGAGGTCGAAACGTA (GRAM/7Fw) and GGTGACAGGATTGGTCTTGTCTTTA (GRAM/161Rv). The amplification product was detected using the following probe: TCAGGCCCCCTCAAAGCCGAG [5']Fam [3']BHQ-1 (GRAM/52/+). To quantify the absolute number of M copies in the samples, a titration curve was done using serial dilutions of RNA from an in vitro-transcribed plasmid containing the M sequence. 

### LDH release assay

Myoblasts and myotubes were infected at a MOI of 30 pfu/plated cell. At 8, 48, 72, 96 or 144 hPI, supernatants were assessed for LDH enzymatic activity using the LDH Cytotoxicity detection kit (Roche). For each time point, non-infected cells were used as a negative control, and lysed non-infected cells as a positive control of LDH release. For cell lysis, 5µl of lysis buffer (Roche) were added in a control well. After 10 min of incubation, supernatants were harvested and clarified by centrifugation at 500g for 5 min. 10µl of each supernatant were diluted in 50µl PBS, and 60µl of the dye / catalyst mix (Roche) were added. After 20 min of incubation at room temperature, 30µl of the stop solution (Roche) were added and the absorbance at 490nm was measured. The percentage of LDH release, or cell permeabilization index, was then calculated according to the following formula: (A490 infected cells – A490 non infected cells) / (A490 lysed cells – A490 non infected cells). 

### Cytokine production after infection

Culture supernatants of infected myoblasts and myotubes were collected at 8 and 24 hPI, clarified and stored at -80°C. The concentration of 5 cytokines and 1 chemokine (IL-6, IL-8, TNF-α, IFN-α, IFN-γ and MCP-1) was then assessed in supernatants through simultaneous detection using the Luminex technology (Cytokine human Singleplex, Invitrogen), following manufacturer's instructions with half of the recommended amount of each reagent. 

## References

[1] Kuiken T, Taubenberger JK (2008) Pathology of human influenza revisited. Vaccine 26 Suppl 4: D59-D66. doi:10.1016/j.vaccine.2008.07.025. PubMed: 19230162. 19230162 PMC2605683

[2] Hayden FG, Fritz R, Lobo MC, Alvord W, Strober W et al. (1998) Local and systemic cytokine responses during experimental human influenza A virus infection. Relation to symptom formation and host defense. J Clin Invest 101: 643-649. doi:10.1172/JCI1355. PubMed: 9449698. 9449698 PMC508608

[3] Lundberg A (1957) Myalgia cruris epidemica. Acta Paediatr 46: 18-31. doi:10.1111/j.1651-2227.1957.tb08627.x. PubMed: 13410584. 13410584

[4] Agyeman P, Duppenthaler A, Heininger U, Aebi C (2004) Influenza-associated myositis in children. Infection 32: 199-203. PubMed: 15293074. 15293074 10.1007/s15010-004-4003-2

[5] Hu JJ, Kao CL, Lee PI, Chen CM, Lee CY et al. (2004) Clinical features of influenza A and B in children and association with myositis. J Microbiol Immunol Infect 37: 95-98. PubMed: 15181490. 15181490

[6] Chi CY, Wang SM, Lin CC, Wang HC, Wang JR et al. (2008) Clinical features of children infected with different strains of influenza B in southern Taiwan. Pediatr Infect Dis J 27: 640-645. doi:10.1097/INF.0b013e31816be008. PubMed: 18520968. 18520968

[7] Mall S, Buchholz U, Tibussek D, Jurke A, An der Heiden M et al. (2011) A large outbreak of influenza B-associated benign acute childhood myositis in Germany, 2007/2008. Pediatr Infect Dis J 30: e142-e146. doi:10.1097/INF.0b013e318217e356. PubMed: 21753259. 21753259

[8] Yoshino M, Suzuki S, Adachi K, Fukayama M, Inamatsu T (2000) High incidence of acute myositis with type A influenza virus infection in the elderly. Intern Med 39: 431-432. doi:10.2169/internalmedicine.39.431. PubMed: 10830190. 10830190

[9] Takayanagi N, Tokunaga D, Kubota M, Hara K, Saito H et al. (2005) Community-acquired pneumonia with rhabdomyolysis. Nihon Kokyuki Gakkai Zasshi 43: 731-735. PubMed: 16457334. 16457334

[10] Abe M, Higuchi T, Okada K, Kaizu K, Matsumoto K (2006) Clinical study of influenza-associated rhabdomyolysis with acute renal failure. Clin Nephrol 66: 166-170. doi:10.5414/CNP66166. PubMed: 16995338. 16995338

[11] Swaringen JC, Seiler JG III, Bruce RW Jr. (2000) Influenza A induced rhabdomyolysis resulting in extensive compartment syndrome. Clin Orthop Relat Res: 243-249. PubMed: 10853175. 10853175 10.1097/00003086-200006000-00029

[12] Perez-Padilla R, de la Rosa-Zamboni D, Ponce de Leon S, Hernandez M, Quiñones-Falconi F et al. (2009) Pneumonia and respiratory failure from swine-origin influenza A (H1N1) in Mexico. N Engl J Med 361: 680-689. doi:10.1056/NEJMoa0904252. PubMed: 19564631. 19564631

[13] Domínguez-Cherit G, Lapinsky SE, Macias AE, Pinto R, Espinosa-Perez L et al. (2009) Critically Ill patients with 2009 influenza A(H1N1) in Mexico. JAMA 302: 1880-1887. doi:10.1001/jama.2009.1536. PubMed: 19822626. 19822626

[14] To KK, Hung IF, Li IW, Lee KL, Koo CK et al. (2010) Delayed clearance of viral load and marked cytokine activation in severe cases of pandemic H1N1 2009 influenza virus infection. Clin Infect Dis 50: 850-859. doi:10.1086/650581. PubMed: 20136415. 20136415 PMC7107930

[15] Koliou M, Hadjiloizou S, Ourani S, Demosthenous A, Hadjidemetriou A (2010) A case of benign acute childhood myositis associated with influenza A (H1N1) virus infection. Clin Microbiol Infect 16: 193-195. doi:10.1111/j.1469-0691.2009.03064.x. PubMed: 19832721. 19832721

[16] D'Silva D, Hewagama S, Doherty R, Korman TM, Buttery J (2009) Melting muscles: novel H1N1 influenza A associated rhabdomyolysis. Pediatr Infect Dis J 28: 1138-1139. doi:10.1097/INF.0b013e3181c03cf2. PubMed: 19841604. 19841604

[17] Ayala E, Kagawa FT, Wehner JH, Tam J, Upadhyay D (2009) Rhabdomyolysis associated with 2009 influenza A(H1N1). JAMA 302: 1863-1864. doi:10.1001/jama.2009.1582. PubMed: 19887664. 19887664

[18] Carrillo-Esper R, Ornelas-Arroyo S, Pérez-Bustos E, Sánchez-Zúñiga J, Uribe-Esquivel M (2009) Rhabdomyolysis and acute renal failure in human influenza A H1N1 mediated infection. Gac Med Mex 145: 519-521. PubMed: 20077871. 20077871

[19] Tosun MS, Ertekin V, Orbak Z (2010) Rhabdomyolysis-induced Acute Renal Failure Associated with 2009 Influenza A (H1N1) Virus Infection in a Child with Crigler-Najjar Syndrome. J Emerg Med. 10.1016/j.jemermed.2010.05.087 20828972

[20] Chen SC, Liu KS, Chang HR, Lee YT, Chen CC et al. (2010) Rhabdomyolysis following pandemic influenza A (H1N1) infection. Neth J Med 68: 317-319. PubMed: 20739729. 20739729

[21] Gutierrez RL, Ellis MW, Decker CF (2010) Rhabdomyolysis and pandemic (H1N1) 2009 pneumonia in adult. Emerg Infect Dis 16: 565. doi:10.3201/eid1603.091818. PubMed: 20202448. 20202448 PMC3322045

[22] Lai CC, Wang CY, Lin HI (2010) Rhabdomyolysis and acute kidney injury associated with 2009 pandemic influenza A(H1N1). Am J Kidney Dis 55: 615. doi:10.1053/j.ajkd.2010.01.002. PubMed: 20189052. 20189052

[23] Parikh M, Dolson G, Ramanathan V, Sangsiraprapha W (2010) Novel H1N1-associated rhabdomyolysis leading to acute renal failure. Clin Microbiol Infect 16: 330-332. doi:10.1111/j.1469-0691.2010.03185.x. PubMed: 20132248. 20132248

[24] Rubín E, De la Rubia L, Pascual A, Domínguez J, Flores C (2010) Benign acute myositis associated with H1N1 influenza A virus infection. Eur J Pediatr 169: 1159-1161. doi:10.1007/s00431-010-1178-7. PubMed: 20213303. 20213303

[25] Morrison TE, Whitmore AC, Shabman RS, Lidbury BA, Mahalingam S et al. (2006) Characterization of Ross River virus tropism and virus-induced inflammation in a mouse model of viral arthritis and myositis. J Virol 80: 737-749. doi:10.1128/JVI.80.2.737-749.2006. PubMed: 16378976. 16378976 PMC1346871

[26] Ozden S, Huerre M, Riviere JP, Coffey LL, Afonso PV et al. (2007) Human muscle satellite cells as targets of Chikungunya virus infection. PLOS ONE 2: e527. doi:10.1371/journal.pone.0000527. PubMed: 17565380. 17565380 PMC1885285

[27] Kumar K, Guirgis M, Zieroth S, Lo E, Menkis AH et al. (2011) Influenza myocarditis and myositis: case presentation and review of the literature. Can J Cardiol 27: 514-522. doi:10.1016/j.cjca.2011.03.005. PubMed: 21652168. 21652168

[28] Mason W, Keller E (1975) Letter: Acute transient myositis with influenza-like illness. J Pediatr 86: 813-814. doi:10.1016/S0022-3476(75)80384-2. PubMed: 1133667. 1133667

[29] Greco TP, Askenase PW, Kashgarian M (1977) Postviral myositis: myxovirus-like structures in affected muscle. Ann Intern Med 86: 193-194. doi:10.7326/0003-4819-86-2-193. PubMed: 835943. 835943

[30] Gamboa ET, Eastwood AB, Hays AP, Maxwell J, Penn AS (1979) Isolation of influenza virus from muscle in myoglobinuric polymyositis. Neurology 29: 1323-1335. doi:10.1212/WNL.29.10.1323. PubMed: 384294. 384294

[31] Farrell MK, Partin JC, Bove KE (1980) Epidemic influenza myopathy in Cincinnati in 1977. J Pediatr 96: 545-551. doi:10.1016/S0022-3476(80)80864-X. PubMed: 7359257. 7359257

[32] Kessler HA, Trenholme GM, Harris AA, Levin S (1980) Acute myopathy associated with influenza A/Texas/1/77 infection. Isolation of virus from a muscle biopsy specimen. JAMA 243: 461-462. doi:10.1001/jama.1980.03300310049022. PubMed: 7351766.7351766

[33] Kessler HA, Trenholme GM, Vogelzang NJ, Patterson R, Semel JD et al. (1983) Elevated creatine phosphokinase levels associated with influenza A/Texas/1/77 infection. Scand J Infect Dis 15: 7-10. PubMed: 6844879. 6844879 10.3109/inf.1983.15.issue-1.02

[34] Ruff RL, Secrist D (1982) Viral studies in benign acute childhood myositis. Arch Neurol 39: 261-263. doi:10.1001/archneur.1982.00510170003001. PubMed: 7073542. 7073542

[35] Bove KE, Hilton PK, Partin J, Farrell MK (1983) Morphology of acute myopathy associated with influenza B infection. Pediatr Pathol 1: 51-66. doi:10.3109/15513818309048284. PubMed: 6687269. 6687269

[36] Gomes M, Muller K, Busch H, Uhl M, Kelly T et al. (2006) An unusual cause of acute rhabdomyolysis. Rheumatology (Oxf) 45: 643-644. doi:10.1093/rheumatology/kel093. 16543385

[37] Baska RE, Frost MD (1977) Acute postinfectious crural myalgia in children. South Med J 70: 419-420. doi:10.1097/00007611-197704000-00011. PubMed: 850807. 850807

[38] Wakabayashi Y, Nakano T, Kikuno T, Ohwada T, Kikawada R (1994) Massive rhabdomyolysis associated with influenza A infection. Intern Med 33: 450-453. doi:10.2169/internalmedicine.33.450. PubMed: 7949648. 7949648

[39] Partin JC, Partin JS, Schubert WK, Jacobs R, Saalfeld K (1976) Isolation of influenza virus from liver and muscle biopsy specimens from a surviving case of Reye's syndrome. Lancet 2: 599-602. PubMed: 61342. 61342 10.1016/s0140-6736(76)90667-x

[40] Ru YX, Li YC, Zhao Y, Zhao SX, Yang JP et al. (2011) Multiple organ invasion by viruses: pathological characteristics in three fatal cases of the 2009 pandemic influenza A/H1N1. Ultrastruct Pathol 35: 155-161. doi:10.3109/01913123.2011.574249. PubMed: 21657817. 21657817

[41] Davis LE, Kornfeld M, Daniels RS, Skehel JJ (2000) Experimental influenza causes a non-permissive viral infection of brain, liver and muscle. J Neurovirol 6: 529-536. doi:10.3109/13550280009091953. PubMed: 11175325. 11175325

[42] Davis LE, Kornfeld M (2001) Experimental influenza B viral myositis. J Neurol Sci 187: 61-67. doi:10.1016/S0022-510X(01)00526-3. PubMed: 11440746. 11440746

[43] Klavinskis LS, Patterson S, Whiting PJ, Wilcox HN, Oxford JS et al. (1985) Infection of cultured human muscle cells by influenza virus. J Gen Virol 66 ( 11): 2335-2345. doi:10.1099/0022-1317-66-11-2335. PubMed: 3903046. 3903046

[44] Armstrong CL, Miranda AF, Hsu KC, Gamboa ET (1978) Susceptibility of human skeletal muscle culture to influenza virus infection. I. Cytopathology and immunofluorescence. J Neurol Sci 35: 43-57. doi:10.1016/0022-510X(78)90101-6. PubMed: 624960. 624960

[45] Servidei S, Miranda AF, Gamboa ET (1987) Infectivity of influenza B virus in cultured human muscle. Acta Neuropathol 73: 67-76. doi:10.1007/BF00695504. PubMed: 3300131. 3300131

[46] Li IW, Chan KH, To KW, Wong SS, Ho PL et al. (2009) Differential susceptibility of different cell lines to swine-origin influenza A H1N1, seasonal human influenza A H1N1, and avian influenza A H5N1 viruses. J Clin Virol 46: 325-330. doi:10.1016/j.jcv.2009.09.013. PubMed: 19801200. 19801200

[47] Nicholls JM, Chan RW, Russell RJ, Air GM, Peiris JS (2008) Evolving complexities of influenza virus and its receptors. Trends Microbiol 16: 149-157. doi:10.1016/j.tim.2008.01.008. PubMed: 18375125. 18375125

[48] Marino M, Scuderi F, Provenzano C, Bartoccioni E (2011) Skeletal muscle cells: from local inflammatory response to active immunity. Gene Ther 18: 109-116. doi:10.1038/gt.2010.124. PubMed: 20927136. 20927136

[49] Davis LE (2010) Neurologic and muscular complications of the 2009 influenza A (H1N1) pandemic. Curr Neurol Neurosci Rep 10: 476-483. doi:10.1007/s11910-010-0135-1. PubMed: 20697982. 20697982

[50] Lazarowitz SG, Goldberg AR, Choppin PW (1973) Proteolytic cleavage by plasmin of the HA polypeptide of influenza virus: host cell activation of serum plasminogen. Virology 56: 172-180. doi:10.1016/0042-6822(73)90296-1. PubMed: 4795670. 4795670

[51] Suelves M, López-Alemany R, Lluís F, Aniorte G, Serrano E et al. (2002) Plasmin activity is required for myogenesis in vitro and skeletal muscle regeneration in vivo. Blood 99: 2835-2844. doi:10.1182/blood.V99.8.2835. PubMed: 11929773. 11929773

[52] Pan HY, Yamada H, Chida J, Wang S, Yano M et al. (2011) Up-regulation of ectopic trypsins in the myocardium by influenza A virus infection triggers acute myocarditis. Cardiovasc Res 89: 595-603. doi:10.1093/cvr/cvq358. PubMed: 21084314. 21084314 PMC3028976

[53] Walther T, Karamanska R, Chan RW, Chan MC, Jia N et al. (2013) Glycomic analysis of human respiratory tract tissues and correlation with influenza virus infection. PLOS Pathog 9: e1003223 PubMed: 23516363. 23516363 10.1371/journal.ppat.1003223 PMC3597497

[54] Nagaraju K, Casciola-Rosen L, Rosen A, Thompson C, Loeffler L et al. (2000) The inhibition of apoptosis in myositis and in normal muscle cells. J Immunol 164: 5459-5465. PubMed: 10799913. 10799913 10.4049/jimmunol.164.10.5459

[55] Hays AP, Gamboa ET (1977) Influenza infections of denervated muscle in mice. 11th World Neurology Congress Amsterdam.

[56] Decary S, Mouly V, Hamida CB, Sautet A, Barbet JP et al. (1997) Replicative potential and telomere length in human skeletal muscle: implications for satellite cell-mediated gene therapy. Hum Gene Ther 8: 1429-1438. doi:10.1089/hum.1997.8.12-1429. PubMed: 9287143. 9287143

[57] Matrosovich M, Matrosovich T, Garten W, Klenk HD (2006) New low-viscosity overlay medium for viral plaque assays. Virol J 3: 63. doi:10.1186/1743-422X-3-63. PubMed: 16945126. 16945126 PMC1564390

[58] Rudnicka D, Feldmann J, Porrot F, Wietgrefe S, Guadagnini S et al. (2009) Simultaneous cell-to-cell transmission of human immunodeficiency virus to multiple targets through polysynapses. J Virol 83: 6234-6246. doi:10.1128/JVI.00282-09. PubMed: 19369333. 19369333 PMC2687379

